# Evaluation of Expanded Criteria Donors Using the Kidney Donor Profile Index and the Preimplantation Renal Biopsy

**DOI:** 10.3389/ti.2022.10056

**Published:** 2022-06-06

**Authors:** F. Villanego, L. A. Vigara, J. M. Cazorla, J. Naranjo, L. Atienza, A. M. Garcia, M. E. Montero, M. C. Minguez, T. Garcia, A. Mazuecos

**Affiliations:** ^1^ Department of Nephrology, Hospital Universitario Puerta del Mar, Cadiz, Spain; ^2^ Department of Pathology, Hospital Universitario Puerta del Mar, Cadiz, Spain

**Keywords:** kidney transplantation, expanded criteria donors, kidney donor profile index, preimplantation biopsy, graft survival

## Abstract

The increasing comorbidity of kidney transplant (KT) donors make it necessary to develop scores to correctly assess the quality of kidney grafts. This study analyzes the usefulness of the preimplantation biopsy and the Kidney Donor Profile Index (KDPI) as indicators of KT survival from expanded criteria donors (ECD). Retrospective study of KT in our center between January 2010 to June 2019 who received a kidney from an ECD and underwent a preimplantation biopsy. 266 KT were included. Graft survival was categorized by KDPI quartiles: Q1 = 86%, Q2 = 95%, Q3 = 99% and Q4 = 100%. KT from KDPI Q1 presented better survival (*p* = 0.003) and Q4 donors had worse renal function (*p* = 0.018) and poorer glomerular filtration rate (3rd month; *p* = 0.017, 1st year; *p* = 0.010). KT survival was analyzed according to KDPI quartile and preimplantation biopsy score simultaneously: Q1 donors with biopsy score ≤3 had the best survival, especially comparing against Q3 with a biopsy score >3 and Q4 donors (*p* = 0.014). In multivariable analysis, hyaline arteriopathy, glomerulosclerosis, and KDPI Q4 were predictors for graft survival. High KDPI and a greater histological injury in the preimplantation biopsy, especially glomerular and vascular lesions, were related to a higher rate of KT loss from ECD.

## GRAPHICAL ABSTRACT

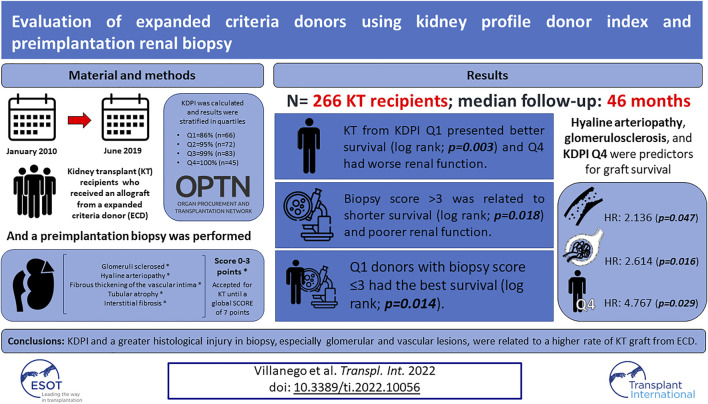

## Introduction

The shortage of kidneys for transplantation has led to the increased use of suboptimal donors. These changes in the demographics of kidney transplant (KT) donors make it necessary to develop tools to assess the suitability of the grafts [1].

Usually, KT viability was determined according to the United Network for Organ Sharing (UNOS) criteria and donors were identified as standard criteria donors (SCD) or expanded criteria donors (ECD) [2]. However, this classification does not adequately reflect the kidney donor’s quality [3,4].

In the last years, several scales have been developed to measure the prognosis of the KT trying to eliminate the dichotomy of SCD versus ECD. The Kidney Donor Profile Index (KDPI) was developed in 2014 by the American Transplantation Registry. It gives a score from 0% to 100% which summarizes the risk of graft failure and it is calculated using 10 donor factors [5]. KDPI is not validated in Spain, but some publications in our country relate the KDPI to renal graft survival [6-9].

Preimplantation biopsy has been used to evaluate the kidney graft, mostly in ECD [10]. There are several scoring systems, such as the Pirani-Remuzzi score or the Maryland Aggregate Pathology index [11,12]. Spanish guidelines for evaluating KT biopsies have been published previously and to date, the acceptance of a kidney from an ECD has been based almost exclusively on the preimplantation biopsy [13]. However, it still has a controversial role in assessing the viability of the renal graft [14,15].

Our main purpose is to analyze the value of preimplantation biopsy and the KDPI in our setting as indicators of KT graft survival from ECD. As a secondary objective, we analyzed the renal graft function and its relationship to the KDPI score and the histological findings in a preimplantation biopsy.

## Methods

### Design and Study Population

We present a retrospective cohort study of KT patients at Puerta del Mar Hospital between 01/01/2010 and 01/06/2019 who received a KT from an ECD (60 years and older and those aged 50–59 years who meet at least two of the following conditions: serum creatinine >1.5 mg/dl, cerebrovascular accident as a cause of death, or hypertension) [3]. We perform a preimplantation biopsy in all kidney grafts from ECD. All patients had a minimum follow-up of 1-year post-KT.

All recipients received immunosuppressive induction with basiliximab or thymoglobuline (5 daily doses of 1 mg/kg, adjusted according to lymphocyte count). Maintenance immunosuppression included tacrolimus (trough level 5–10 ng/ml), mycophenolate mofetil (1,000–2,000 mg/day), and prednisone (5 mg/day).

### Biopsies Assessment

Graft biopsies were obtained by renal wedge during the bench surgery from a representative part of the graft, avoiding scars. KT biopsies were analyzed by four expert pathologists. All of these biopsies had more than 25 glomeruli. The ECD KT with biopsies from other centers were excluded. The samples were processed fresh and tissue was frozen immediately using methyl butane which was cooled in liquid nitrogen. Subsequently, the cuts were made in the cryostat, and stained with rapid hematoxylin-eosin staining. The results are obtained in 15 min approximately.

The biopsy score was calculated following the Spanish protocol for preimplantation biopsy, based on the Remuzzi score. Five parameters were evaluated: glomerular sclerosis, myointimal elastosis, hyaline arteriopathy, interstitial fibrosis, and tubular atrophy. They were scored from 0 to 3, depending on the degree of injury. A global score ≥7 or a score of 3 in any of the first 3 histological compartments is considered unfavorable for transplantation and graft should be discarded [13].

### Variables

We analyzed donor and KT recipient variables, and estimated glomerular filtration rate 3 months and 1 year after KT. The score obtained in each individual histological component and the cumulative score for pathological lesions of the preimplantation biopsy were collected. Kidney graft survival was defined as the time from transplant to graft failure, censoring for death with a functioning graft. Deceased patients with a functioning graft were considered as lost to follow-up. Glomerular filtration rate was estimated by the Modification of Diet in Renal Disease (MDRD-4) [16]. The KDPI score was calculated using the formula on the Organ Procurement and Transplantation Network website [17].

### Statistical Analysis

Continuous variables are presented as mean and standard deviation or median and interquartile range as appropriate; categorical variables as frequencies and percentages. Categorical variables were compared using Fisher exact test or Chi-square test, and continuous variables using the Student’s t-test, U Mann-Whitney, or ANOVA, according to normality and number of groups. For multiple comparisons in continuous variables, Bonferroni correction was conducted. Normality was analyzed by the Kolmogorov-Smirnov test.

The KDPI was analyzed as an absolute value and stratified according to quartiles. The biopsy score was stratified according to the mean value of the assessment scale (score = 3). Graft survival categorized by KDPI quartile and biopsy score were plotted using the Kaplan-Meier method and compared between groups by log-rank test. Pairwise testing over strata was performed if > 2 groups were compared in survival analysis.

To identify risk factors associated with graft failure univariable and multivariable analysis was performed using Cox regression. Pretransplant variables related to graft survival, KDPI, and biopsy score were included in the multivariable analysis as well as other covariates based on the criterion of *p*-value <0.1 in the univariable analysis. Several models were performed to analyze the global biopsy score, the different histological compartments, and the KDPI as a continuous and a categorical variable according to quartiles. In the models that included KDPI, donor variables already evaluated in the score (such as age and diabetes) were excluded.

Values significant *p* < 0.05 were considered. The statistical analysis was performed with SPSS v.25.

## Results

In the study period, 720 KT were performed in our center, of which 83 corresponded to living KT donors. In 267 no biopsy was performed and in 104 KT the biopsy was processed in another center. Finally, 266 KT met the criteria and were included. The median follow-up was 46 months.

Baseline characteristics of donors and recipients are presented in Table 1. Grafts were stratified by quartiles based on the KDPI score: Q1 = 86% (*n* = 66); Q2 = 95% (*n* = 72); Q3 = 99% (*n* = 83); Q4 = 100% (*n* = 45). The median KDPI was 95% and the median biopsy score was 2 points. Four donors had acute kidney injury at the time of donation. However, they did not present greater histological scores compared to donors with normal renal function (score 3 [2.25,3] vs. score 2 [2,3]; *p* = 0.479).

**TABLE 1:** Characteristics of KT donors and recipients included.

Donors	*n* = 161
Sex female, n (%)	68 (42.2)
Age (years), median [IQR]	66 [60,70]
HBP, n (%)	72 (44.7)
DM, n (%)	27 (16.8)
Brain death donor, n (%)	132 (81.9)
Smoking, n (%)	49 (30.4)
Serum creatinine (mg/dl), mean ± SD	0.8 ± 0.3
Height (cm), mean ± SD	165.2 ± 7.8
Weight (kg), ±SD	79.1 ± 13.5
KDPI quartile	
Q1: 86%, n (%)	66 (24.8)
Q2: 95%, n (%)	72 (27.1)
Q3: 99%, n (%)	83 (31.2)
Q4: 100%, n (%)	45 (16.9)
KDPI (%), median [IQR]	95 [86,99]
Biopsy score, median [IQR]	2 [2,3]
**Recipients**	***n* = 266**
Sex female, n (%)	97 (36.5)
Age (years), median [IQR]	62 [52.75, 68]
Etiology of CKD	
DM, n (%)	35 (13.1)
HBP, n (%)	18 (6.8)
GN, n (%)	42 (15.8)
Others, n (%)	79 (29.7)
Unknown, n (%)	92 (34.6)
Retransplant, n (%)	22 (8.3)
RRT pre-KT	
HD/PD/preemptive KT, n (%)	192 (72.2)/63 (23.7)/11 (4.1)
RRT time (months), median [IQR]	17 [8,28]
HCV+, n (%)	7 (2.6)
**Transplant**	
CIT (minutes), median [IQR]	1195 [946,1390]
DGF, n (%)	110 (41.3)
Q1, n (%)	25 (37.8)
Q2, n (%)	30 (41.6)
Q3, n (%)	25 (30.1)
Q4, n (%)	30 (66.6)
Primary graft non-function, n (%)	14 (5.2)
Q1, n (%)	2 (3)
Q2, n (%)	2 (2.7)
Q3, n (%)	4 (4.8)
Q4, n (%)	6 (13)

IQR, interquartile range; HBP, high blood pressure; DM, diabetes mellitus; SD, standard deviation; KDPI, kidney donor profile index; DGF, delayed graft function; Q, quartile; CKD, chronic kidney disease; GN, glomerulonephritis; KT, kidney transplant; HD, hemodialysis; PD, peritoneal dialysis; RRT, renal replacement therapy; HCV, hepatitis C virus.

### Kidney Graft Function

Renal function was worse at 3 months and 1-year post-KT, especially in grafts with a Q4 KDPI (Table 2). Similarly, kidneys with biopsy scores>3 presented worse eGFR in the 3rd month (−11.3 ml/min; *p* = 0.017) and after the first year post-KT (−8.4 ml/min; *p* = 0.010) (Table 2).

**TABLE 2:** Renal function at 3 months and 1 year after kidney transplantation. (A) Renal function according to KDPI quartile. (B) Renal function according to biopsy score.

MDRD at 3rd month^a^, mean ± SD	Q1 (*n* = 47)	Q2 (*n* = 54)	Q3 (*n* = 46)	Q4 (*n* = 38)	*p*-value
	45.8 ± 16.5^ade^	40.1 ± 20.3^bdf^	37.6 ± 21.5^cef^	27.3 ± 18.3^abc^	a < 0.001
					b = 0.005
					c = 0.036
					d = 0.087
					e = 0.012
					f = 0.297
MDRD at 1st year^a^, mean ± SD	Q1 (*n* = 47)	Q2 (*n* = 50)	Q3 (*n* = 43)	Q4 (*n* = 36)	*p*-value
	46.8 ± 19.3^ade^	39.6 ± 22^bdf^	38.0 ± 24.3^cef^	28.1 ± 19.3^abc^	a<0.001
					b = 0.039
					c = 0.910
					d = 1.000
					e = 0.195
					f = 0.213
MDRD at 3rd month, mean ± SD	Biopsy score ≤ 3 (*n* = 196)		Biopsy score > 3 (*n* = 43)		*p*-value
	43.6 ± 16.6		32.3 ± 20.3		0.017
MDRD at 1st year, mean ± SD	Biopsy score ≤ 3 (*n* = 186)		Biopsy score > 3 (*n* = 40)		
	40.1 ± 22.1		31.7 ± 21.8		0.010

a ANOVA test: *p* < 0.001. Comparison between KDPI quartiles (Bonferroni correction).

MDRD at 3rd month: ^a^Q1 vs. Q4, ^b^Q2 vs. Q4, ^c^Q3 vs. Q4, ^d^Q1 vs. Q2, ^e^Q1 vs. Q3, ^f^Q2 vs. Q3 and MDRD at 1st year: ^a^Q1 vs. Q4, ^b^Q2 vs. Q4, ^c^Q3 vs. Q4, ^d^Q1 vs. Q2, ^e^Q1vs. Q3, ^f^Q2 vs. Q3.

MDRD = 0 was considered in patients reinitiating hemodialysis.

Q, quartile; MDRD, modification of diet in renal disease; SD, standard deviation.

### Kidney Graft Survival

**FIGURE 1:**
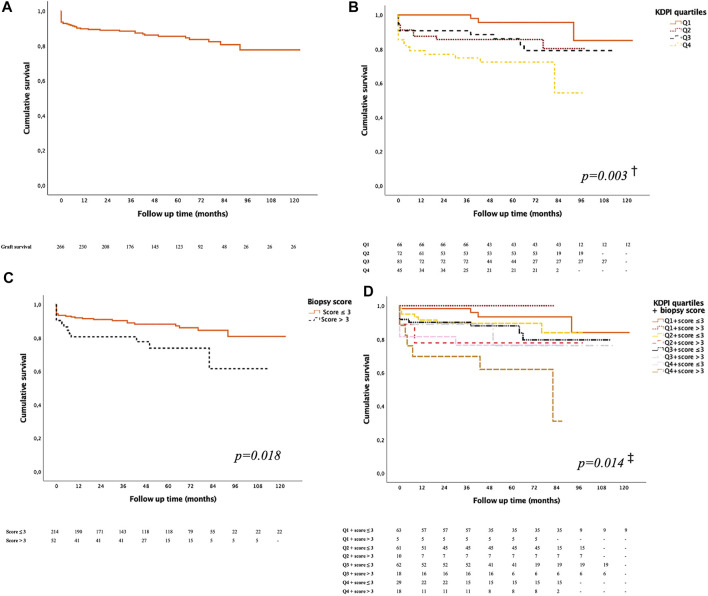
Kidney graft survival function. **(A)** All kidney transplant patients. **(B)** According to the KDPI quartile. ^†^Q1 vs. Q4; *p* = 0.001, Q1 vs. Q2; *p*= 0.012, Q1 vs. Q3; *p* = 0.043; the rest of pairwise com*p*arisons were not significant: Q2 vs. Q3; *p* = 0.876, Q2 vs. Q4; *p* = 0.110, Q3 vs. Q4; *p* = 0.192. **(C)** According to the score of the preimplantation biopsy. **(D)** According to the combination of the KDPI quartile and the score of the preimplantation biopsy. ^‡^Q1 & score ≤3 vs. Q2 & score ≤3; *p* = 0.019, Q1 & score ≤3 vs. Q3 & score >3; *p* = 0.023, Q1 & score ≤3 vs. Q4 & score ≤3; *p* = 0.009, Q1 & score ≤3 vs. Q4 & score >3; *p* < 0.001, the rest of *p*airwise com*p*arisons were not significant: Q1 & score ≤3 vs. Q1 & score >3; *p* = 0.728, Q1 & score ≤3 vs. Q2 & score >3; *p* = 0.117, Q1 & score ≤3 vs. Q3 & score ≤3; *p* = 0.125, Q1 & score >3 vs. Q2 & score ≤3; *p* = 0.376, Q1 & score >3 vs. Q2 & score >3; *p* = 0.398, Q1 & score >3 vs. Q3 & score ≤3; *p* = 0.425, Q1 & score >3 vs. Q3 & score >3; *p* = 0.238, Q1 & score >3 vs. Q4 & score ≤3; *p* = 0.252, Q1 & score >3 vs. Q4 & score >3; *p* = 0.175, Q2 & score ≤3 vs. Q2 & score >3; *p* = 0.935, Q2 & score ≤3 vs. Q3 & score ≤3; *p* = 0.583, Q2 & score ≤3 vs. Q3 & score >3; *p* = 0.669, Q2 & score ≤3 vs. Q4 & score ≤3; *p* = 0.310, Q2 & score ≤3 vs. Q4 & score >3; *p* = 0.089, Q2 & score >3 vs. Q3 & score ≤3; *p* = 0.676, Q2 & score >3 vs. Q3 & score >3; *p* = 0.875, Q2 & score >3 vs. Q4 & score ≤3; *p* = 0.612, Q2 & score >3 vs. Q4 & score >3; *p* = 0.357, Q3 & score ≤3 vs. Q3 & score >3; *p* = 0.449, Q3 & score ≤3 vs. Q4 & score ≤3; *p* = 0.308, Q3 & score ≤3 vs. Q4 & score >3; *p* = 0.073, Q3 & score >3 vs. Q4 & score ≤3; *p* = 0.948, Q3 & score >3 vs. Q4 & score >3; *p* = 0.574, Q4 & score ≤3 vs. Q4 vs. score >3; *p* = 0.479.

Death-censored graft survival was 89.8% at 1 year and 85.4% at 5 years post-KT (Figure 1A). Regarding the survival of the allograft by quartile of KDPI, kidneys from donors in the lowest quartile presented better outcomes (*p* = 0.001). Pairwise testing did not show differences between other groups (Figure 1B).

We compared graft survival according to preimplantation biopsy score: score ≤3, *n* = 214 (80.5%) vs. score >3, *n* = 52 (19.5%). KT with a biopsy score >3 presented worse survival (*p* = 0.018) (Figure 1C). In addition, these biopsies corresponded to higher KDPI donors: the mean of the KDPI for score ≤3 was 86.4 ± 17.7%, vs. 93.8 ± 11.4%. for score >3 (*p* < 0.001).

Finally, KT survival was compared according to the quartile of KDPI and preimplantation biopsy scores simultaneously. Q1 donors with less histological injury (score ≤3) had the best survival rate, especially compared against Q3 with a biopsy score >3 and Q4 donors (*p* = 0.014) (Figure 1D).

We analyzed graft survival by histological compartments, comparing the absence (score 0) and the presence of histological injury (scores 1 and 2). The absence of glomerulosclerosis and hyaline arteriopathy were associated with a better graft survival (*p* = 0.005 and *p* = 0.034), but not the histological injury in the rest of the compartments (Supplementary Table S1).

### Cox Regression Analysis

In the univariable analysis, donor age, diabetic donor, biopsy score >3, KDPI, glomerulosclerosis, and hyaline arteriopathy were related to a higher rate of graft loss (Table 3).

**TABLE 3:** Univariable and multivariable Cox regression analysis for death-censored graft failure.

Univariable analysis		
	HR (95% CI)	*p*-value
Female donor	1.643 (0.882–3.059)	0.118
Donor age	1.055 (1.014–1.098)	0.008
HBP donor	0.795 (0.388–1.630)	0.531
DM donor	2.654 (1.277–5.516)	0.009
Smoking donor	0.761 (0.355–1.633)	0.484
Non-heart beating donor	1.095 (0.458–2.616)	0.839
KDPI	1.034 (1.003–1.066)	0.029
Female recipient	1.074 (0.566–2.037)	0.828
Recipient age	1.031 (0.997–1.065)	0.071
Time of RRT	1.000 (1.000–1.001)	0.194
Cold ischemia time	1.000 (0.999–1.001)	0.986
Biopsy score > 3	2.173 (1.120–4.218)	0.022
Glomerulosclerosis ≥1	2.305 (1.033–5.143)	0.041
Hyaline arteriopathy ≥1	1.349 (1.090–5.059)	0.029
Myointimal elastosis ≥1	1.524 (0.463–5.021)	0.489
Tubular atrophy ≥1	0.661 (0.307–1.420)	0.289
Interstitial fibrosis ≥1	1.304 (0.589–2.886)	0.512
**Multivariable analysis**		
Model 1		
Cold ischemia time	1.000 (1.000–1.001)	0.576
KDPI	1.032 (0.996–1.069)	0.081
Recipient age	0.998 (0.957–1.039)	0.910
Time of RRT	1.000 (1.000–1.001)	0.181
Biopsy score > 3	1.719 (0.855–3.456)	0.128
Model 2		
Cold ischemia time	1.000 (1.000–1.001)	0.710
Recipient age	0.987 (0.945–1.031)	0.548
Time of RRT	1.000 (1.000–1.001)	0.210
Biopsy score > 3	1.414 (0.680–2.940)	0.354
KDPI Q2^a^	2.503 (0.738–8.492)	0.141
KDPI Q3^a^	3.136 (0.842–11.680)	0.088
KDPI Q4^a^	6.684 (1.583–28.229)	0.010
Model 3		
Cold ischemia time	1.000 (1.000–1.001)	0.593
KDPI	1.030 (0.994–1.068)	0.101
Recipient age	0.996 (0.956–1.038)	0.858
Time of RRT	1.000 (1.000–1.001)	0.236
Hyaline arteriopathy ≥1	2.322 (1.124–4.794)	0.023
Glomerulosclerosis ≥1	2.861 (1.330–6.154)	0.007
Model 4		
Cold ischemic time	1.000 (0.999–1.001)	0.722
Recipient age	0.988 (0.947–1.032)	0.595
Time of RRT	1.000 (1.000–1.001)	0.233
Hyaline arteriopathy ≥1	2.136 (1.010–4.516)	0.047
Glomerulosclerosis ≥1	2.614 (1.193–5.729)	0.016
KDPI Q2^a^	2.217 (0.650–7.559)	0.203
KDPI Q3^a^	3.111 (0.850–11.388)	0.086
KDPI Q4^a^	4.767 (1.177–19.315)	0.029

a Reference KDPI Q1.

KDPI, kidney donor profile index; RRT, renal replacement therapy; HR, hazard ratio; CI, confidence interval.

Models performed in the multivariable analysis are shown in Table 3. In the model that included KDPI and biopsy score, only KDPI was at the limit of statistical significance as a predictor of KT loss (*p* = 0.081) (Model 1). When KDPI was analyzed as quartiles, Q4 was an independent risk factor for graft survival (*p* = 0.010) (Model 2). Replacing score biopsy for glomerulosclerosis and hyaline arteriopathy, the presence of these lesions was related to a worse graft survival (*p* = 0.007; *p* = 0.023) (Model 3). Finally, when we included KDPI quartiles and the score of glomerulosclerosis and hyaline arteriopathy, glomerulosclerosis (*p* = 0.016), hyaline arteriopathy (*p* = 0.047), and Q4 KDPI (*p* = 0.029) remained as independent predictors for kidney graft survival (Model 4).

## Discussion

This study presents one of the biggest cohorts and with the largest follow-up that analyzes the efficacy of the graft preimplantation biopsy and the KDPI simultaneously in the evaluation of the ECD. Both variables are necessary for the assessment of non-optimal grafts. Additionally, we have identified a very high-risk group of donors, who are those with a KDPI greater than 99% and a biopsy score >3 points.

The usefulness of preimplantation biopsy as a predictive tool for graft survival has not been validated yet and some studies discuss its value for the assessment of the quality of organs from ECD [15,18-20]. In our case, all the grafts had a preimplantation biopsy and only those with a global score <7 were accepted. KT with a score <3 had better survival and allograft function, reflecting the potential value of the biopsy. However, in multivariable analysis, when donor clinical variables were included, the global score did not associate with graft survival. Previous studies did not find survival differences when comparing KT with mild and moderated lesions in preimplantation biopsy, so the discard of an allograft should not be done exclusively according to the histological analysis [21,22].

We observed that glomerulosclerosis and hyaline arteriopathy was related to shorter graft survival. Some groups have defended the value of glomerulosclerosis as the main parameter in the evaluation of the preimplantation biopsy, showing that a percentage of glomerular sclerosis >20% is associated with a worse graft evolution [23-25]. Bröcker et al. stated that patients with hyaline arteriopathy usually had worse renal function [26]. Our results remained even when KDPI was included in the model, so not only determining the global biopsy score is important but which histological compartments are most affected.

Regarding a typical frozen sections’ biopsy analysis, subtle findings such as interstitial fibrosis and tubular atrophy, thickening of capillary walls, mesangial cellularity, and histological lesions related to diabetes and other comorbidities are often more difficult to identify compared to correctly fixed and stained sections such as microwave paraffin embedding [15,24,27]. In our case, the use of the freezing technique may have underestimated the degree of some histological injuries. However, in all grafts, a wedge biopsy was performed to ensure the proper quality of the sample. New ultra-fast freezing techniques seem to offer better results with a higher quality of the histological sample, although their use has not yet become widespread in kidney donor biopsies [28]. Our results show that glomerulosclerosis and hyaline arteriopathy should be considered the main histological compartments in biopsies processed with the most commonly used freezing methods.

Because of the controversy about the efficacy of histologic evaluation for predicting graft survival, the KDPI has been implemented in the United States as an effective system for evaluating the quality of deceased donors [5]. Along with our experience, kidney allografts from donors with a lower KDPI had better survival. Additionally, we observed a negative relationship between KDPI and graft function. In the United States, a kidney with a KDPI >85% is considered suboptimal and it is likely rejected. However, there are many transplant centers that currently utilize high KDPI kidneys [29,30]. In our study, the median KDPI score was 95%, but one-year graft survival was 89.8% and 85.4% at 5 years, which is higher than reported in other series with a lower KDPI [31]. Notwithstanding, the KDPI is a tool developed by the American Transplantation Registry, so its usefulness in Spain is limited by the difficulty to extrapolate this score to other countries with different healthcare systems and transplant programs [6,9].

Due to the technical limitations of the preimplantation biopsy and the lack of accuracy of KDPI, it seems reasonable the combination both variables for the assessment of the ECD. We analyzed simultaneously KDPI and biopsy scores in an ECD cohort and we identified a group at high risk of graft failure: KDPI greater than 99% and biopsy score >3. However, a KT with a very high KDPI score that does not present these lesions in the biopsy can offer an acceptable medium-term survival (5-year graft survival: 78.7%), especially through an old-for-old allocation program or for high estimated post-transplant survival (EPTS) recipients [32]. On the other hand, dual KT could be the best choice to improve the results of transplantation with grafts with both high KDPI and high biopsy scores [33].

Our study presents several limitations. It is a retrospective, single-center study, with the limitations that inherently may exist in data collection. Second, biopsies were not re-evaluated retrospectively by a single pathologist in order to reduce the interobserver variability. However, only a limited group of expert pathologists analyzed the biopsies. Therefore, to avoid further biases, we excluded kidneys with biopsies analyzed in other centers where there could be differences in the preparation and interpretation of the histological samples. In third place, according to our protocol, the grafts with a very high biopsy score were not implanted, so we cannot be sure what function they might have had. Fourth, the use of the frozen-section analysis has drawbacks that have already been discussed. Last, when we analyzed the KDPI and the biopsy score as continuous in the multivariate models, we found no significant differences. However, when both variables were stratified, survival inequalities were observed. These divergences in the results may be due to the multicollinearity between both variables.

In conclusion, the KDPI and a greater histological injury in the preimplantation biopsy, especially glomerular and vascular lesions, were related to a higher rate of KT graft loss coming from ECD. Both parameters were related to graft function and survival. As long as a kidney donor evaluation index more adapted to our country is not available as well as more rapid and precise histological techniques, we suggest that both the clinical and histological variables should be considered together in the pretransplant assessment of ECD with a high KDPI.

## Data Availability

The data underlying this article will be shared on reasonable request to the corresponding author.

## Abbreviations

DGF, delayed graft function; ECD, expanded criteria donors; EPTS, estimated post-transplant survival; KDPI, kidney donor profile index; KT, kidney transplantation; MDRD-4, Modification of Diet in Renal Disease-4; SCD, standard criteria donor; UNOS, United Network for Organ Sharing.

## References

[1] National Transplant Organization. Donation and Transplant Activity Reports [Internet] (2020). Available from: http://www.ont.es/infesp/Memorias/Actividad_de_Donaci%C3%B3n_y_Trasplante_Renal_2019.pdf (Accessed June 12, 2021).

[2] Port FK Bragg-Gresham JL Metzger RA Dykstra DM Gillespie BW Young EW Donor Characteristics Associated with Reduced Graft Survival: an Approach to Expanding the Pool of Kidney Donors1. Transplantation (2002) 74:1281–6. 10.1097/00007890-200211150-00014 12451266

[3] Wohlfahrtova M Viklicky O. New Strategies for Evaluating the Quality of Kidney Grafts from Elderly Donors. Transplant Rev (2015) 29(4):212–8. 10.1016/j.trre.2015.04.002 25971422

[4] Lee APK Abramowicz D. Is the Kidney Donor Risk Index a Step Forward in the Assessment of Deceased Donor Kidney Quality? Nephrol Dial Transpl (2015) 30(8):1285–90. 10.1093/ndt/gfu304 25282158

[5] Israni AK Salkowski N Gustafson S Snyder JJ Friedewald JJ Formica RN New National Allocation Policy for Deceased Donor Kidneys in the United States and Possible Effect on Patient Outcomes. J Am Soc Nephrol (2014) 25(8):1842–8. 10.1681/asn.2013070784 24833128 PMC4116061

[6] Calvillo-Arbizu J Pérez-Valdivia MA Gentil-Govantes MA Castro-de-la-Nuez P Mazuecos-Blanca A Rodríguez-Benot A Does the Kidney Donor Profile Index (KDPI) Predict Graft and Patient Survival in a Spanish Population? Nefrología (English Edition) (2018) 38(6):587–95. 10.1016/j.nefroe.2018.06.014 30243494

[7] del Moral Martín RMG Retamero Díaz JA Cava Molina M Cobacho Tornel BM Bravo Soto J Osuna Ortega A Validation of KDRI/KDPI for the Selection of Expanded Criteria Kidney Donors. Nefrología (English Edition) (2018) 38(3):297–303. 10.1016/j.nefroe.2017.12.003 29132985

[8] Pascual J Pérez-Sáez MJ. Kidney Donor Profile Index: Can it Be Extrapolated to Our Environment? Nefrología (English Edition) (2016) 36(5):465–8. 10.1016/j.nefro.2016.05.005 27550146

[9] Arias-Cabrales C Pérez-Sáez MJ Redondo-Pachón D Buxeda A Burballa C Bermejo S Usefulness of the KDPI in Spain: A Comparison with Donor Age and Definition of Standard/expanded Criteria Donor. Nefrología (2018) 38(5):503–13. 10.1016/j.nefro.2018.03.003 29884503

[10] Dare AJ Pettigrew GJ Saeb-Parsy K. Preoperative Assessment of the Deceased-Donor Kidney. Transplantation (2014) 97(8):797–807. 10.1097/01.tp.0000441361.34103.53 24553618

[11] Remuzzi G Cravedi P Perna A Dimitrov BD Turturro M Locatelli G Long-term Outcome of Renal Transplantation from Older Donors. N Engl J Med (2006) 354(4):343–52. 10.1056/nejmoa052891 16436766

[12] Chen K Guntur G Stalam T Okonkwo N Drachenberg C Goussous N Deceased-Donor Kidney Biopsy Scoring Systems for Predicting Future Graft Function: A Comparative Study. Transplant Proc (2021) 53:906–12. 10.1016/j.transproceed.2020.09.002 33358418

[13] Serón D Anaya F Marcén R del Moral RG Martul EV Alarcón A Guidelines for Indicating, Obtaining, Processing and Evaluating Kidney Biopsies. Nefrologia (2008) 28(4):385–96. 18662146

[14] Navarro MD López-Andréu M Rodríguez-Benot A Ortega-Salas R Morales MLA López-Rubio F Significance of Preimplantation Analysis of Kidney Biopsies from Expanded Criteria Donors in Long-Term Outcome. Transplantation (2011) 91(4):432–9. 10.1097/TP.0b013e318204bdd7 21157404

[15] Wang CJ Wetmore JB Crary GS Kasiske BL. The Donor Kidney Biopsy and its Implications in Predicting Graft Outcomes: A Systematic Review. Am J Transpl (2015) 15(7):1903–14. 10.1111/ajt.13213 25772854

[16] Levey AS Bosch JP Lewis JB Greene T Rogers N Roth D. A More Accurate Method to Estimate Glomerular Filtration Rate from Serum Creatinine: A New Prediction Equation. Ann Intern Med (1999) 130:461–70. 10.7326/0003-4819-130-6-199903160-00002 10075613

[17] Organ Procurement and Transplantation Network. KDPI Calculator [Internet] (2020). Available from https://optn.transplant.hrsa.gov/resources/allocation-calculators/kdpi-calculator/ (Accessed June 17, 2021).

[18] Amenábar JJ Camacho JA Gómez-Larrambe N Visus T Pijoan JI González del Tánago J Prognostic Utility of Preimplantation Kidney Biopsy from Deceased Older Donors in First Year post-transplant Renal Function. Nefrología (English Edition) (2016) 36(1):33–41. 10.1016/j.nefro.2015.10.009 26698928

[19] Teixeira AC Ferreira E Marques MG Rodrigues L Santos L Romãozinho C Pretransplant Biopsy of Marginal Kidneys: Is it Necessary? Transplant Proc (2019) 51(5):1585–9. 10.1016/j.transproceed.2019.02.023 31155197

[20] Colussi G Casati C Colombo VG Camozzi MLP Salerno FR. Renal Transplants from Older Deceased Donors: Is Pre-implantation Biopsy Useful? A Monocentric Observational Clinical Study. World J Transplant (2018) 8(4):110–21. 10.5500/wjt.v8.i4.110 30148077 PMC6107519

[21] Hofer J Regele H Böhmig GA Gutjahr G Kikić Ž Mühlbacher F Pre-implant Biopsy Predicts Outcome of Single-Kidney Transplantation Independent of Clinical Donor Variables. Transplantation (2014) 97(4):426–32. 10.1097/01.tp.0000437428.12356.4a 24285339

[22] Foss A Heldal K Scott H Foss S Leivestad T Jørgensen PF Kidneys from Deceased Donors More Than 75 Years Perform Acceptably after Transplantation. Transplantation (2009) 87(19):1437–41. 10.1097/TP.0b013e3181a4ebd2 19461478

[23] Escofet X Osman H Griffiths DFR Woydag S Adam Jurewicz W. The Presence of Glomerular Sclerosis at Time Zero Has a Significant Impact on Function after Cadaveric Renal Transplantation. Transplantation (2003) 75(3):344–6. 10.1097/01.TP.0000044361.74625.E7 12589156

[24] Gaber LW Moore LW Alloway RR Amiri MH Vera SR Gaber AO. Glomerulosclerosis as a Determinant of Posttransplant Function of Older Donor Renal Allografts. Transplantation (1995) 60:334–8. 10.1097/00007890-199508270-00006 7652761

[25] Randhawa PS Minervini MI Lombardero M Duquesnoy R Fung J Shapiro R Biopsy of Marginal Donor Kidneys: Correlation of Histologic Findings with Graft Dysfunction1. Transplantation (2000) 69(7):1352–7. 10.1097/00007890-200004150-00024 10798753

[26] Bröcker V Schubert V Scheffner I Schwarz A Hiss M Becker JU Arteriolar Lesions in Renal Transplant Biopsies. Am J Pathol (2012) 180(5):1852–62. 10.1016/j.ajpath.2012.01.038 22464889

[27] Carpenter D Husain SA Brennan C Batal I Hall IE Santoriello D Procurement Biopsies in the Evaluation of Deceased Donor Kidneys. Clin J Am Soc Nephrol (2018) 13(12):1876–85. 10.2215/CJN.04150418 30361336 PMC6302333

[28] Renne SL Redaelli S Paolini B. Cryoembedder, Automatic Processor/stainer, Liquid Nitrogen Freezing, and Manual Staining for Frozen Section Examination: A Comparative Study. Acta Histochem (2019) 121(6):761–4. 10.1016/j.acthis.2019.05.002 31078257

[29] Gupta A Francos G Frank A Shah A. KDPI Score Is a strong Predictor of Future Graft Function: Moderate KDPI (35 - 85) and High KDPI (> 85) Grafts Yield Similar Graft Function and Survival. Clin Nephrol (2016) 86(10):175–82. 10.5414/CN108858 27616757

[30] Rege A Irish B Castleberry A Vikraman D Sanoff S Ravindra K Trends in Usage and Outcomes for Expanded Criteria Donor Kidney Transplantation in the United States Characterized by Kidney Donor Profile Index. Cureus (2016) 8(11):e887. 10.7759/cureus.887 28018757 PMC5179248

[31] Sánchez-Escuredo A Sagasta A Revuelta I Rodas LM Paredes D Musquera M Histopathological Evaluation of Pretransplant Donor Biopsies in Expanded Criteria Donors with High Kidney Donor Profile index: a Retrospective Observational Cohort Study. Transpl Int (2017) 30(10):975–86. 10.1111/tri.12966 28403541

[32] Organ Procurement and Transplantation Network. A Guide to Calculating and Interpreting the Estimated Post-Transplant Survival (EPTS) Score Used in the Kidney Allocation System (KAS) [Internet] (2021). Available from: https://optn.transplant.hrsa.gov/media/1511/guide_to_calculating_interpreting_epts.pdf (Accessed June 17, 2021).

[33] Snanoudj R Timsit M-O Rabant M Tinel C Lazareth H Lamhaut L Dual Kidney Transplantation: Is It Worth It? Transplantation (2017) 101(3):488–97. 10.1097/TP.0000000000001508 27748703

