# Supplementary material for: Evaluation of Expanded Criteria Donors Using the Kidney Donor Profile Index and the Preimplantation Renal Biopsy
Source: Transpl Int. 2022 Jun 6;35:10056. doi: 10.3389/ti.2022.10056 (PMC9207180; doi:10.3389/ti.2022.10056)
Supplement: Supplementary file 1 [file Table1.pdf]

SUPPLEMENTAL MATERIAL

Table S1: Survival of the grafts according to the score of each histological compartment.

Histological compartment		P-value†
**Glomerulosclerosis**	Score 0 vs. 1-2	0.005
**Hyaline arteriopathy**	Score 0 vs. 1-2	0.034
**Myointimal elastosis**	Score 0 vs. 1-2	0.471
**Tubular atrophy**	Score 0 vs. 1-2	0.223
**Interstitial fibrosis**	Score 0 vs. 1-2	0.557

† *Log-rank test*
